# Account of Diseases in an Eastern District of London, from the 20th of September to the 20th of October, 1801

**Published:** 1801-11

**Authors:** 


### *Account of Diseases in an Eastern District of London, from the 20th of September to the 20th of October,* 1801.

| | |
|---|---|
| ACUTE DISEASES. | |
| Typhus | 22 |
| Pneumonia | 2 |
| Dyfenteria | 15 |
| Rheumatifmus Acutus | 2 |
| CHRONIC DISEASES. | |
| Peripneumonia Notha | 4 |
| Phthifis Pulmonalis | 2 |
| Tuffis | 10 |
| Tuffis et Dyfpnœa | 7 |
| Pleurodyne | 3 |
| Hepatitis Chronica | 1 |
| Hydrothorax | 2 |
| Anafarca | 3 |
| Afcites | 1 |
| Diarrhœa | 10 |
| Hæmorrhois | 4 |
| Tenefmus | 6 |
| Amenorrhœa | 3 |
| Menorrhagia | 5 |
| Leucorrhœa | 4 |
| Hypochondriafis | 1 |
| Vertigo | 3 |
| Paralyfis | 1 |
| Vomitus | 4 |
| Prolapfus Vaginæ | 1 |
| Herpes | 5 |
| Rheumatifmus Chronicus | 15 |
| PUERPERAL DISEASES. | |
| Low Puerperal Fever | 3 |
| Menorrhagia Lochialis | 1 |
| Maftodynia | 3 |
| Dyfuria | 1 |
| INFANTILE DISEASES. | |
| Febris Mefenterica | 1 |
| Herpes | 4 |
| Tinea Capitis | 2 |
| Diarrhœa | 12 |

The fever which has long prevailed, and the influence of which has been fo extenfively diffufed, ftill continues. The fymptoms attending it are very fimilar to thofe which have lately been defcribed.

Thofe violent affections of the brain, which have formed fo important a characteriftic of the difeafe for a confiderable time, are lefs frequent; and at prefent, difeafe of the ftomach and bowels feems to be a more common attendant upon this fever. This occurs under various forms, and in different degrees. A moderate diarrhœa occurring at an early ftage of the difeafe, has generally proved falutary, and has frequently afforded a juft prognofis of a favourable termination of the difeafe: But when at a more advanced period, evacuations from the bowels have increafed, have affumed a dark appearance, and have exhaled a fœtid odour, they muft be viewed as fymptomatic of difeafe and danger, rather than as affording the hope of any critical relief. It by no means, however, follows from hence, that fuch evacuations are to be checked, whilft the prefence of this offenfive matter is an indication of difeafe; the removal of it may prove the means of relief; and therefore to correct and difcharge what is fo offenfive to the inteftine and to the conftitution, is furely a more rational practice than to detain it.

Befide thefe affections of the bowels, which may be confidered as fymptomatic, there have been others which have conftituted the original difeafe: A large number of dyfenteries have lately occurred, and fome of them have proved very obftinate.

This difeafe, as it is well known, ufually occurs at this feafon of the year; and as a diarrhœa frequently prevails at the fame time, owing probably, in fome inftances, to a larger quantity of fruit being eaten, thefe difeafes are too often confounded. The patient complains of pain in his bowels, accompanied with a large number of ftools; and before any medical affiftance is requefted, every domeftic medicine calculated to ftop a purging is adminiftered: But when the quantity and kind of difcharge from the inteftines are examined, it proves, that although the inclination to have a ftool has been very frequent, yet the difcharge has been very fmall, and this confifting rather of mucus, or mucus-blood, than of fæces. A confiderable degree of fever ufually accompanies this difeafe; and the frequent inclination to go to ftool, and the tenefmus which fucceeds it, are a fource of conftant uneafinefs. This difeafe is to be traced to a fpafmodic ftricture in the courfe of the large inteftines, by which fæces are detained, and, confequently, the cure muft be attempted by relaxing the fpafm, and evacuating the fæces. Opium may be adminiftered as an antifpafmodic, but its exhibition fhould be immediately fucceeded by that of a brifk cathartic. In the treatment of moft of the cafes referred to in the lift, Pulv. ipec. camp. from 10 to 15 grains, was preferred to any other opiate, and cryft. tart. from 2 to 4 drachms, with 6 or 8 grains of fcammony, generally anfwered the purpofe of difcharging a confiderable quantity of fæces, which was followed by an abatement of the moft urgent fymptoms.

